# Biomechanical Stability of Tibia Plateau Fracture Treatment: Conventional vs. Finite Element‐Based Preoperative Planning

**DOI:** 10.1002/jor.70188

**Published:** 2026-04-02

**Authors:** Sabrina Sandriesser, Robert Pätzold, Simon Comtesse, Lea Sommerhalder, Thomas Zumbrunn, Arvind von Keudell, Benjamin Stäudle, Peter Augat

**Affiliations:** ^1^ Institute for Biomechanics BG Unfallklinik Murnau Murnau Germany; ^2^ Institute for Biomechanics Paracelsus Medical University Salzburg Austria; ^3^ Department of Trauma and Orthopaedic Surgery BG Unfallklinik Murnau Murnau Germany; ^4^ Institute for Biomechanics ETH Zurich Zurich Switzerland; ^5^ CustomSurg AG Zurich Switzerland; ^6^ Department of Orthopaedic Surgery Brigham and Women's Hospital, MassGeneral Brigham Harvard Medical School Boston USA; ^7^ Department of Orthopaedic Surgery and Traumatology; University of Copenhagen, Department of Clinical Medicine Copenhagen University Hospital Bispebjerg Denmark; ^8^ Rimasys GmbH Cologne Germany

## Abstract

Tibial plateau fractures are complex injuries requiring anatomical reduction and stable fixation to restore joint congruency and function. Digital tools, including CT reconstructions, computer‐assisted implant planning, and finite element (FE) modeling, have the potential to improve fixation strategies. This experimental study investigated whether FE‐based preoperative planning enhances the stability of tibial plateau fracture fixation compared with conventional planning, assessing construct stiffness, load to failure, and fracture stability under physiologic loading. Twelve human cadaveric lower limbs (78 ± 10 years) with induced Schatzker IV fractures were randomized to conventional (*n* = 6) or FE‐based planning (*n* = 6). In the FE group, fragment reduction, screw trajectories, and implant positioning were optimized via computational modeling and guided intraoperatively by individual targeting guides. Conventional planning used standard CT visualization. All specimens were fixed using a medial locking plate and tested under axial loading, including stiffness measurement and progressively increasing cyclic loading until failure. Plate and screw positioning did not clearly differ between approaches, however FE‐based planning promoted more consistent locking screw utilization and more frequent individual screw usage. FE‐based planning yielded higher load to failure (1050 ± 535 N vs. 442 ± 226 N, *p* = 0.041), more cycles to failure (10,100 ± 5400 vs. 4100 ± 2400; *p* = 0.046), and more symmetrical construct stiffness. After adjustment for anatomical variations, differences in failure load were no longer statistically significant. Tibial plateau widening during loading was comparable between groups. These findings suggest that FE‐based planning can enhance construct stability and reduce fixation asymmetry. Further clinical validation is needed to determine whether these benefits translate into improved outcomes.

## 1 Introduction

Tibia plateau fractures (TPFs) represent complex intra‐articular injuries that require precise anatomical reduction and stable internal fixation to restore joint congruency and mechanical function of the knee [1, 2]. TPFs are commonly caused by high energy trauma in younger adults, or by low‐energy mechanisms in elderly individuals with osteoporotic bone [3]. Achieving optimal outcomes in these cases remains a surgical challenge due to the intricate nature of the fracture morphology and the biomechanical demands during weight‐bearing activities [1]. Proper surgical management is crucial, as inadequate fracture reduction and insufficient fixation can lead to complications such as joint instability, post‐traumatic osteoarthritis, and impaired limb function [4]. Common aseptic complications include malalignment, malunion, or even non‐union of fracture fragments, and hardware‐related problems. In surgically treated TPFs, unsatisfactory reduction has been reported in up to 30% of cases, with clinical outcomes being strongly influenced by surgical experience [5].

To improve the surgical outcomes, preoperative planning of complex TPFs is gaining more and more importance, and recent advances have been reported [6]. As a first step, CT‐based 3D reconstructed models help surgeons better visualize the complex, multi‐fragmentary fracture pattern [7]. In a second step, these models can be used to design patient‐specific drill guides for optimized screw placement or can be 3D printed to serve as an intraoperative guidance [8, 9].

Recent advances in computational modeling, particularly finite element analysis, offer a promising approach for enhancing preoperative surgical planning. FE analysis allows for biomechanical simulation of various fixation strategies and implant configurations based on patient‐specific anatomy and loading conditions [10]. More specifically, different plate positions and screw configurations can be evaluated preoperatively to help the surgeon select the most stable and anatomically appropriate construct. Emerging research has demonstrated the value of FE analysis in assessing the fixation stability of TPFs, providing a valuable tool for optimizing preoperative planning in the management of these complex injuries [11, 12, 13].

The objective of this experimental study was to investigate and compare the biomechanical performance of tibia plateau fracture fixation using conventional surgical planning versus FE‐based preoperative planning. We hypothesized that FE‐based preoperative planning would result in a more stable fracture fixation, in terms of construct stiffness, load to failure, and plateau widening under physiological loads, when tested on realistic fracture models seen in clinical practice.

## 2 Materials and Methods

Eighteen fresh frozen left lower extremities from human cadavers were obtained through a commercial body donor program (Science Care, Phoenix, AZ, US) for inclusion in this study. Bone mineral density (BMD) in mg/ccm was assessed at the proximal tibia based on a CT scan with a calibration phantom. Preserving the knee joint, specimens with intact soft tissue were dissected mid‐femur and distally above the ankle joint to enable a standardized machine‐based fracture process via axial loading [14], generating Schatzker IV tibial plateau fractures with lateral depression (Rimasys GmbH, Cologne, Germany). A CT scan of the lower extremity confirmed that the induced fractures were comparable with those seen in clinical practice [15].

**Figure 1:**
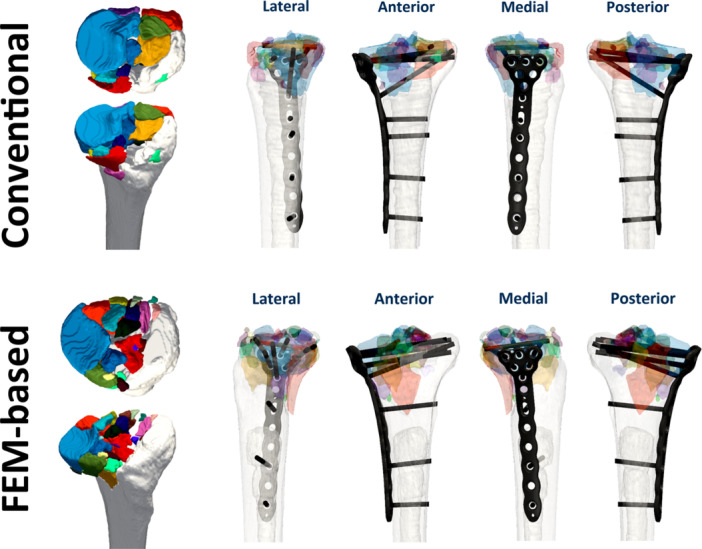
Representative matched pair of fractures in top and anterior oblique views, demonstrating high morphological similarity. The upper‐row specimen was allocated to the conventional group, with plate and screw placement planned and performed conventionally. The lower‐row specimen was allocated to the FE‐based group, with plate and screw placement digitally planned and FE‐optimized.

From the initial set of 18 experimentally induced fractures in left human cadaveric tibiae, the 12 fractures demonstrating the greatest morphological similarity were selected by two orthopaedic trauma surgeons. These fractures (*n* = 12) were subsequently matched into pairs by two different fellowship‐trained orthopaedic trauma surgeons in conjunction with a senior biomedical engineer, based on the number, location, and size of the fracture fragments (Figure 1). Particular emphasis was placed on the morphology and extent of the lateral fracture component (complete fracture, posterolateral rim fracture, or depression only) and on the presence and extent of a medial split component. Within each matched pair, fractures were then randomly allocated to either the conventional or the FE‐based [12] fixation group.

### 2.1 Specimen Preparation

**Figure 2:**
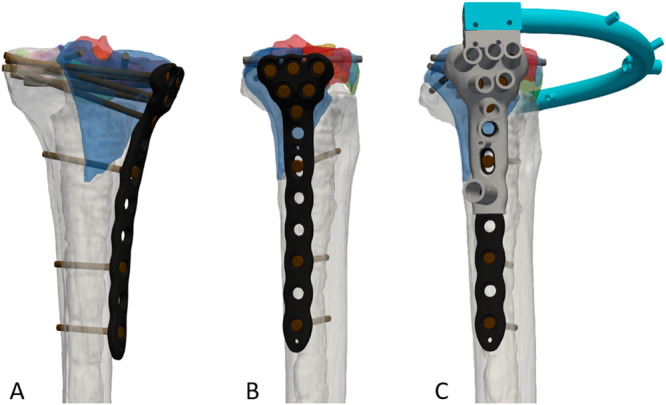
(A) FE‐based fragment reduction and optimized implant position from posterior view, (B) medial view, (C) and medial view with 3D printed targeting guides for the plate and additional anterior–posterior screws.

The specimens were kept frozen at −20° Celsius and were thawed for 24 h before preparation. For both groups, a medial locking plate with variable angle locking screw options was used (Pangea, proximal medial tibia plate, left, 8‐hole, Stryker GmbH, Selzach, Switzerland) and both conventional (non‐locking) as well as locking screws were available for plate fixation. For additional fragment fixation, fully threaded cannulated screws (ASNIS, 4.0 mm, Stryker AG, Selzach Switzerland) were used. The utilization of the different screw types was noted and analyzed. Specimen pairs were randomly assigned to one of six senior surgeons, with each surgeon operating on one specimen from each group within the same matched pair. For the conventional group, surgical fixation was guided by conventional surgical planning based on visual inspection of preoperative CT scans. In the FE‐based group, preoperative planning included optimal fragment reduction and screw length selection based on virtual 3D models, while the optimal implant position and screw orientations were iteratively optimized based on finite element modeling [12]. Fragment reduction and implant fixation were guided by customized and sample‐specific 3D printed bone models and targeting devices that fitted on top of the plate and provided the predefined directions of locking and individual anterior‐posterior screws (Figure 2). Intraoperatively, the intended implant fit and position were verified by fluoroscopy.

After implantation, the tibia was dissected by removing the femur and all soft tissue. Measured from the most proximal portion of the tibia, the shaft was cut at a length of 17 cm by an oscillating saw. Ensuring horizontal alignment of the tibia plateau, the specimens were embedded in an aluminum pot using a three‐component casting resin (Fast Cast Resin 010 A/B + Filler DT 082‐1, Gößl + Pfaff GmbH, Germany). To avoid embedding of the implant, the plate and the screw tips were covered with modeling clay. During preparation and testing, the samples were kept moist using gauze swabs that were sprayed with saline solution.

### 2.2 Mechanical Setup

**Figure 3:**
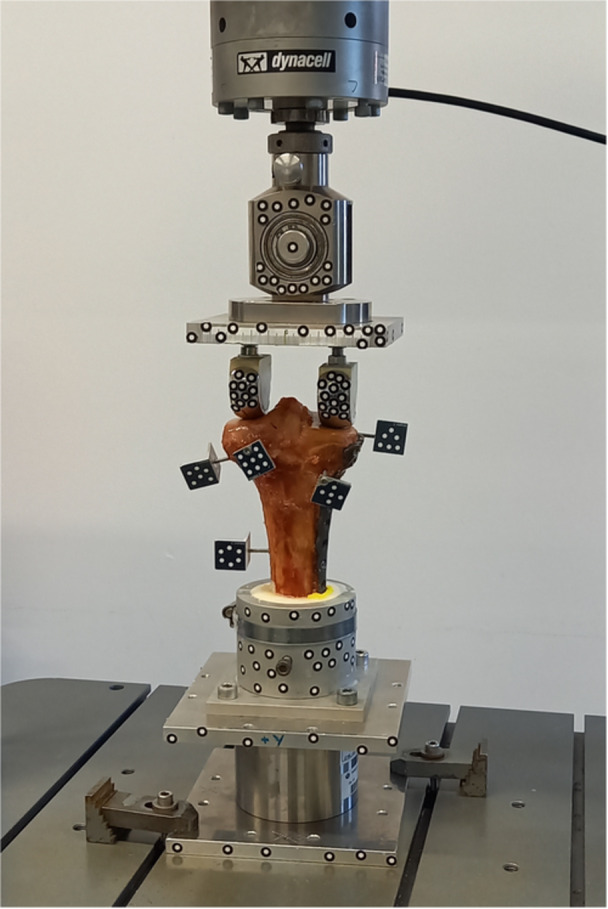
Posterior view of the test setup with load application via two femoral hemicondyles mounted on a rocker to minimize constraint forces. Marker points were attached to the test fixtures and the fracture fragments to evaluate interfragmentary movements.

Axial loading of the fixated tibial plateau fractures was performed using an electrodynamic testing machine (Instron E3000, Instron GmbH, Germany) (Figure 3). Stainless steel prosthetic femoral hemicondyles were mounted via a rocker to the load cell and actuator. For each specimen, the tibial plateau width was measured between the medial and lateral margins. The rocker was positioned on the tibial plateau, with the spacing of the steel hemicondyles adjusted to ensure centering over the medial and lateral compartments. This alignment was intended to replicate physiological knee joint loading conditions, in which 60% of the applied force is transmitted through the medial plateau and 40% through the lateral plateau, thereby creating realistic load distribution during testing [16, 17].

The load protocol was divided into three steps. First, the construct was preconditioned with sinusoidal axial load from 10 to 50 N at 1 Hz for 100 cycles. This was followed by four quasi‐static displacement‐controlled loading ramps at a velocity of 2 mm/min from 10 to 200 N, where the fourth ramp was used to measure axial construct stiffness. In a third step, cyclic sinusoidal loading started from 10 to 50 N at a frequency of 1 Hz. Every 500 cycles, the maximum load was incrementally increased by 50 N, while the lower load level was kept constant at 10 N. Failure was defined as either a vertical impression of the condyles exceeding 3 mm into the tibia plateau or a varus/valgus malalignment greater than 5° [18].

Fragment movement was measured by marker points that were tracked by an optical 3D motion tracking system (ARAMIS 6 M, Carl Zeiss GOM Metrology GmbH, Germany). Marker flags were placed at each accessible fragment, defined on the CT segmented virtual 3D model, as well as on the lateral and medial hemicondyles, the rocker, and the embedding pot. Pictures were taken at the lower load level of 10 N and at the maximum load for each respective load level. The coordinate system was defined with its origin placed at the bottom center of the embedding pot. The z‐axis was aligned vertically along the axis of the machine actuator, the y‐axis pointed posteriorly, and the x‐axis was directed laterally. Varus/valgus rotation, axial subsidence of the condyles, fragment movement, as well as failure mode, failure load, and cycles to failure were recorded and analyzed.

### 2.3 Data Analysis

To account for variations in specimen size and loading configuration, the medio‐lateral width of the tibial plateau and the distances between the hemicondyles were analyzed for each group. Axial construct stiffness was determined from the axial subsidence of the hemicondyles within the linear portion of the load–displacement curve obtained during the fourth loading ramp. Stiffness values were calculated separately for the medial and lateral tibial plateaus, allowing side‐specific evaluation of construct performance. Failure load and failure cycles were investigated at the respective failure mode of either > 5° varus/valgus malalignment of the condyles in the frontal plane or > 3 mm subsidence of the condyles into the tibia plateau along the vertical axis [18]. Additionally, tibia plateau widening was analyzed based on the change in distance in the transverse axis between the most lateral and most medial tibia plateau fragments at two time points: after 2000 load cycles, which is the maximum load level of the weakest construct (equivalent to 200 N), and at the respective failure of each construct.

Force and stiffness data were tested for normal distribution using Shapiro–Wilk tests, and statistical analysis was conducted using Student's *t*‐tests (SPSS Statistics, version 26, IBM, US). To account for anatomical variation, analysis of covariance (ANCOVA) was performed using the relevant anatomical parameters as covariates when comparing failure loads between groups. Implant configurations between groups were compared by calculating median screw usage and conducting paired Wilcoxon signed‐rank tests. Associations between screw usage and stiffness and failure load were evaluated by Spearman's rank correlation. Values are given as mean and standard deviation. The level of significance was set to 0.05.

## 3 Results

Tibia epiphyseal bone mineral density was comparable between the conventional and the FE‐planned groups (124 ± 37 mg/ccm vs. 116 ± 38 mg/ccm, *p* = 0.743). Likewise, there was no difference in gender distribution (male/female: 4/2 vs. 4/2), age (82 ± 8 years vs. 75 ± 11 years) and BMI (22 ± 7 kg/m^2^ vs 19 ± 3 kg/m^2^) between the groups (*p* > 0.05, respectively). The tibial plateau width was slightly larger in the FE group (80 ± 5 mm) compared to the conventional group (76 ± 5 mm), although the difference was not statistically significant (*p* = 0.16). Because the distance between the hemicondyles could only be adjusted in 5 mm increments, the resulting spacing of the condyles during the mechanical tests was significantly larger in the FE group (52.5 ± 2.5 mm) compared with the conventional group (47.5 ± 2.5 mm; *p* = 0.01).

The total number of plate screws ranged from 7 to 11 screws in the conventional group and 8 to 9 screws in the FE‐based group (median 9 screws for both groups; *p* = 0.7). There was a trend toward increased locking screw utilization in the FE‐based group (median 8, range 7–8) compared to the conventional group (median 6.5; range: 5–10; *p* = 0.4). Notably, while the FE analysis consistently recommended at least one additional cannulated screw for fragment fixation (median 2, range 1–3), the surgeons applied individual screws only in half of the cases (median 0.5; range 0–3; *p* = 0.1). Among the stability measures, only medial construct stiffness (plate side) was associated with screw usage, increasing with the total number of plate screws (*R* = 0.74; *p* = 0.006). Neither the number of locking screws nor individual screw usage was associated with construct stiffness or failure load (*p* > 0.1).

**Figure 4:**
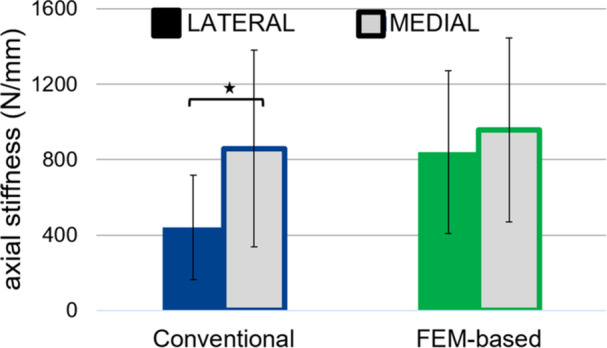
Axial construct stiffness (N/mm) for the conventional and FE‐based groups, for the lateral and the medial tibia plateau separately. The asterisk marks significant differences.

Overall, construct stiffness was slightly larger for the FE planned group (898 ± 487 N/mm) compared with the conventional group (649 ± 428 N/mm; *p* = 0.37). In the conventional group, stiffness on the medial side where the plate was placed, was almost twice that of the lateral side opposite the plate (mean difference: 418 ± 315 N/mm, *p* = 0.023) In contrast, the FE‐based group showed nearly symmetrical stiffness between the plated medial side and the lateral side (mean difference: 120 ± 261 N/mm, *p* = 0.313) (Figure 4).

**Figure 5:**
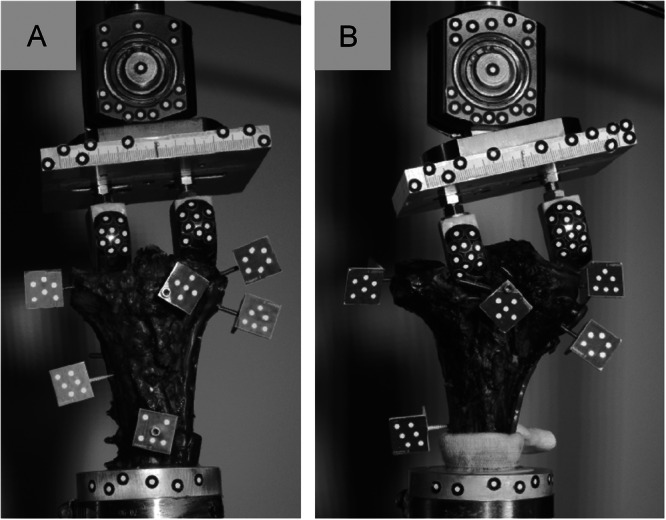
Posterior view of the left specimens with medial plate osteosynthesis. Representative failure modes: (A) Subsidence of the lateral hemicondyle greater than 3 mm, (B) abrupt valgus malalignment greater than 5°.

In eleven specimens, failure occurred by subsidence of the lateral hemicondyle by more than 3 mm, while one specimen in the conventional group failed due to valgus malalignment greater than 5° (Figure 5). The FE‐based group failed at significantly higher loads (1050 ± 535 N) compared to the conventional group (442 ± 226 N, *p* = 0.041). Correspondingly, the number of cycles to failure was greater in the FE‐based group (10,050 ± 5366) than in the conventional group (4083 ± 2370) (*p* = 0.046). However, when the observed failure loads were adjusted for tibial plateau width or condyle spacing using analysis of covariance, the group differences were no longer statistically significant (*p* > 0.1).

**Figure 6:**
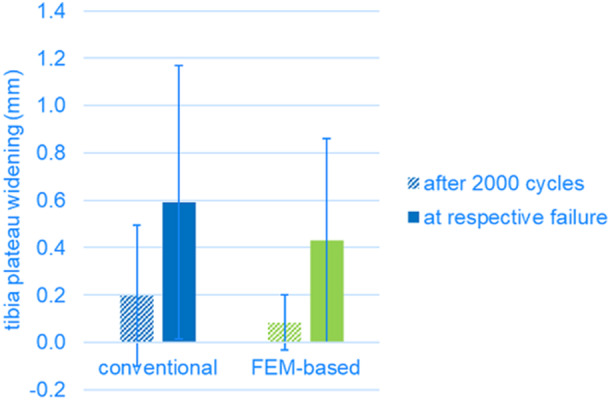
Tibia plateau widening (mm) for the conventional and FE‐based groups. The dashed bars represent plateau widening after 2000 load cycles (equivalent to 200 N). The solid bars represent the plateau widening at the respective failure loads.

Tibia plateau widening after 2000 load cycles (0.20 ± 0.30 mm conventional, 0.08 ± 0.12 mm FE‐based, *p* = 0.448) and at the respective failure loads (0.59 ± 0.58 mm conventional, 0.43 ± 0.43 mm FE‐based, *p* = 0.627) was comparable for both groups (Figure 6).

## 4 Discussion

This study investigated whether finite element model (FE)‐based preoperative planning of fracture fixation offers biomechanical advantages over conventional surgical planning in realistic Schatzker IV tibial plateau fractures with lateral depression. The findings demonstrated that fixation constructs guided by FE‐based planning exhibited higher mechanical stability. This was evidenced by a significantly higher load to failure. Conventional surgical planning resulted in marked asymmetry of construct stiffness, with diminished stiffness on the side opposite the osteosynthesis plate, whereas FE‐based preoperative planning produced a more uniform and symmetrical mechanical construct. In the FE‐based group, medial and lateral stiffness were comparable, indicating a more homogeneous load distribution and improved overall construct stability. Taken together, these findings suggest that FE‐based preoperative planning can mitigate mechanical asymmetries commonly associated with unilateral fixation strategies and may ultimately contribute to improved outcomes in the management of complex TPFs.

The observed benefits align with previous reports that emphasize the importance of patient‐specific and individualized preoperative planning in the management of complex articular fractures [6, 10, 12]. A systematic review on the operative management of foot and ankle fractures agreed on improved surgical outcomes when using 3D printed models for preoperative surgical planning [19]. Our data corroborate the theory that computational planning, including 3D printed models and targeting devices, supports the selection of optimal implant positioning and screw trajectories, ultimately leading to a biomechanically favorable and more stable fixation construct. Although our analysis did not reveal definitive differences in the specific positioning and configuration of plates and screws between the FE‐based and conventional approaches, the quantitative assessment of hardware utilization provides some insights into potential contributing factors. Overall, the FE‐based approach appeared to promote more consistent and strategic implementation of locking screws relative to non‐locking screws. The discrepancy in the number of locking screws used in the FE‐based group compared to the standard group arose from the surgeon's preference for using cortical screws to secure the plate in the tibial shaft. Furthermore, the FE analysis systematically recommended the incorporation of additional cannulated individual screws for interfragmentary fixation, which was less consistently adopted in the conventional approach. In particular, a higher total number of screws in the plate was associated with increased construct stiffness at the plate side (medial). Collectively, these hardware utilization patterns may have contributed to enhanced biomechanical stability of the FE‐planned fracture construct. Further investigation is warranted to determine whether integration of FE‐based recommendations into preoperative planning translates into measurable improvements in biomechanical construct stability and, ultimately, improved clinical outcomes.

A recent study by Assink et al. reported on patient‐specific osteosyntheses and individualized drilling guides for TPFs [20]. Patient‐specific approaches offer the potential for optimized implant positioning and accurate screw placement. Our study confirmed the benefit of optimized screw positioning with the help of patient‐specific 3D printed targeting devices. Such guides help the surgeon to place the screws in predefined directions and support intraoperative decision‐making. Furthermore, the computational model can suggest the optimal amount and trajectory of locking screws to achieve maximum construct stability.

The challenges of treating TPFs, particularly those with multi‐fragmentary patterns, are well‐known. Schatzker IV fractures with lateral depression pose significant surgical challenges due to their complex fracture geometry. Particularly in complex fractures, proper surgical management is crucial to achieve adequate fracture reduction and sufficient fragment fixation [2]. Importantly, enhanced fixation stability may contribute to improved postoperative outcomes by reducing the risk of secondary displacement or complications such as joint instability or post‐traumatic osteoarthritis [4]. Schatzker et al. reported on the management of TPFs and emphasized a good anatomical reduction and stable fragment fixation to restore the weight‐bearing surface and joint congruency, both of which are crucial for enabling early mobilization and promoting faster rehabilitation [1].

Clinical literature reports unsatisfactory reduction rates of up to 30% in surgically treated TPFs, often attributed to inadequate preoperative planning and improper intraoperative decision‐making [5]. FE‐based 3D planning of TPFs represents a promising approach that provides real‐time guidance to implement the preoperative plan into the actual surgery [9, 21]. This can be particularly beneficial for less experienced surgeons, offering an objective framework to guide decision‐making and reduce intraoperative trial and error [22]. Traditionally, conventional planning based on two‐dimensional imaging often insufficiently characterizes the complex three‐dimensional morphology of these fractures. The integration of CT‐based 3D representation and finite element models not only enables improved visualization but also offers predictive insight into the mechanical behavior of different fixation strategies [7, 23]. The results of the current study provide quantitative biomechanical evidence supporting these assumptions, demonstrating that management of TPFs supported by FE‐based preoperative planning exhibits significantly greater resistance to cyclic sinusoidal loading compared to conventional surgical planning.

In biomechanical studies, smooth fracture lines are commonly created using defined and precise osteotomies; however, these simplified fracture models do not accurately represent the complex morphologies and patterns of real fractures [24, 25]. A strength of the current experimental study is that realistic Schatzker IV tibial plateau fractures with lateral depression were generated via axial loading. This standardized machine‐based fracture process ensured the inclusion of realistic fracture patterns seen in clinical practice. In order to guarantee comparability, experienced surgeons paired the specimens according to the fracture patterns, which were then randomly assigned to one of the two groups.

The adapted load application has already been established in a previously published study [26]. Two hemicondyles were mounted on a rocker to guarantee physiologic load distribution. Based on the tibia plateau width, the hemicondyle distance was adjusted for each sample individually, to apply 60% of the load on the medial and 40% on the lateral tibia plateau. Van Rossom et al. demonstrated that during gait, average contact forces were higher on the medial than on the lateral tibia plateau [16]. This established approach guaranteed a physiological load ratio of 60/40 on the tibia plateau, according to the commonly known Miculicz line. However, this sample‐specific setting represents a limitation of the study, as it accentuated preexisting anatomical differences between the groups. The tibial plateaus were wider in the specimens of the FE‐based group, and adjusting the condyle spacing to center the condyles on their respective plateaus resulted in a wider condyle spacing in this group. Such increased spacing may have influenced load induction into the tibial plateau, since larger spans were associated with higher failure loads. Consequently, it cannot be clearly determined whether the observed group differences were attributable to FE planning or to differences in condyle spacing.

Further limitations of this study have to be acknowledged. While the loading conditions applied were designed to mimic physiological weight‐bearing, complex in vivo joint biomechanics caused by muscles and soft tissue interactions could not be considered in this study. In general, biomechanical in vitro studies have the inherent weakness that fracture healing cannot be taken into account. This study included human specimens at a mean age of 78 ± 10 years, representing an elderly population commonly affected by low‐energy fractures of the tibia plateau [3]. Values for bone mineral density were evenly distributed across both groups (*p* = 0.743). Nonetheless, because of the inter‐specimen variability of human donor bone, rather high standard deviations were observed [27]. The study groups in the current study consisted of *n* = 6 samples each, owing to the limited availability of human donor bone and the challenge of reproducibly producing similar types of fractures. A left–right paired‐specimen approach was not feasible, as the fracture creation process—particularly for Schatzker type IV patterns—inevitably introduces specimen‐specific variability that may compromise comparability between paired limbs. A larger sample size might be favorable to ensure adequate statistical power.

Another limitation is that a medial locking plate was used for Schatzker IV fractures with lateral depression. Although this approach reflects a widely accepted and less invasive fixation strategy, dual plating is sometimes preferred in severe or highly unstable fracture patterns to achieve enhanced biomechanical stability. In order to ensure methodological consistency and comparability between groups, all specimens were treated with a medial plate only. This standardized approach represents a clinically relevant worst‐case scenario as the lateral column remains unsupported without a second plate. Notably, under these challenging conditions, the superior biomechanical performance observed in the FE‐based group underscores the value of FE guided preoperative planning. It highlights the ability to optimize screw trajectories and implant configurations to achieve stable fixation, even when more conservative fixation strategies are used. Clinically, this may support the use of medial‐only plating in selected cases, reducing surgical invasiveness while still maintaining construct stability.

Moreover, while the implementation of FE analysis into the surgical workflow seems promising, its adoption may be limited by technical barriers, including the need for specialized software, expertise, and time [28]. For the successful integration of an FE‐based preoperative planning workflow into clinical routine, continued development of user‐friendly interfaces is essential to ensure seamless adoption by the operating surgeon. Furthermore, prospective clinical studies are necessary to evaluate whether the biomechanical advantages demonstrated in this study translate into improved functional and radiological outcomes postoperatively.

In conclusion, this study demonstrates that 3D guided preoperative planning offers biomechanical advantages in the fixation of complex tibia plateau fractures, particularly in terms of unilateral construct stability on the side opposite the osteosynthesis plate. These findings underscore the potential of advanced 3D surgical planning to enhance surgical precision and of finite element modeling to optimize implant placement and improve fixation stability. Nonetheless, these advantages must be interpreted with caution, as differences in condyle spacing between groups may have affected load induction and failure loads. FE‐based planning may ultimately support better clinical outcomes in TPF management, but its routine integration into preoperative workflows requires further technical validation. Clinical superiority should be confirmed through prospective clinical studies.

## Author Contributions

All authors made substantial contributions to the study conception and design. Material preparation and data collection were performed by Sabrina Sandriesser, Robert Pätzold, Simon Comtesse, and Lea Sommerhalder. Data were analyzed by Sabrina Sandriesser, Robert Pätzold and Peter Augat and discussed with all authors. The first draft of the manuscript was written by Sabrina Sandriesser and Peter Augat and was revised by all authors. All authors read and commented on previous versions of the manuscript as well as approved the final manuscript.

## Funding

The authors received no specific funding for this work.

## Ethics Statement

The authors have nothing to report.

## Conflicts of Interest

Simon Comtesse, Lea Sommerhalder and Thomas Zumbrunn are employees of CustomSurg AG, Zurich, Switzerland. Benjamin Stäudle is employee of Rimasys GmbH, Cologne, Germany.

## Data Availability

Data is made available upon reasonable request.
